# Telehealth and Telecare: supporting unpaid carers in Scotland

**Published:** 2012-06-15

**Authors:** Donna Henderson, Anne Conlin

**Affiliations:** Scottish Centre for Telehealth and Telecare, Scotland; Carers Scotland, Scotland

**Keywords:** unpaid carers, awareness raising, education, support, self-management

## Abstract

The Scottish Government Joint Improvement Team (JIT) and the Scottish Centre for Telehealth (SCT), now merged as the Scottish Centre for Telehealth and Telecare (SCTT), published an Education and Training Strategy for Telehealthcare in Scotland in March 2010. In implementing the Strategy’s dedicated Carers Workstream, the SCTT has been working with Carers Scotland and other carer organisations to promote awareness of the benefits of telehealth and telecare in helping to support unpaid carers. Following on from research undertaken by the University of Leeds into the benefits of telecare for carers, developments to date includes the publication of a Training Toolkit for professionals and carer organisations to assist with raising awareness of the benefits of telehealth and telecare specifically for unpaid carers. The toolkit includes outline training programmes, handouts, digital stories and case studies which all adaptable for local use. The presentation will provide an overview of the broader strategic carers’ agenda in relation to telehealth and telecare service delivery, an in-depth look at the Carers and Telehealthcare Training Toolkit and an update on an exciting new technology project to support young carers, being developed in collaboration with Princess Royal Trust for Carers, Glasgow Caledonian and Edinburgh Universities.

Project objectives/deliverables:

1. Work with local authority, health boards and other partners to raise awareness of telehealth and telecare care amongst carers nationally and locally.
2. Identify methods and programme for delivery to ensure that carers have access to appropriate information about access to telehealth and telecare services.
3. Develop core content and supporting materials in carer awareness and telehealth and telecare training to form a Carers and Telehealthcare Training Toolkit.
4. Evaluate impact of Telehealthcare Training Toolkit on staff and carers to inform future development of Toolkit contents.
5. Incorporate telehealth and telecare for carers’ content into core curriculum and CPD modules for health and social care staff.
6. Identify methods and programme for delivery to facilitate the integration of telehealth and telecare needs into carer, community care and health assessment processes.
7. Work with Glasgow Caledonian University and partners to enhance young carers’ access to information and support via a technology solution.

